# Investigation of multilevel data storage in silicon-based polycrystalline ferroelectric tunnel junction

**DOI:** 10.1038/s41598-017-04825-z

**Published:** 2017-07-03

**Authors:** Pengfei Hou, Jinbin Wang, Xiangli Zhong

**Affiliations:** 1 School of Materials Science and Engineering, Xiangtan University, Hunan Xiangtan, 411105 China; 2 Hunan Provincial National Defense Key Laboratory of Key Film Materials & Application for Equipment, Xiangtan University, Hunan Xiangtan, 411105 China; 3 Key Laboratory of Low-dimensional Materials and Application Technology, Xiangtan University, Hunan Xiangtan, 411105 China

## Abstract

Multilevel data ferroelectric tunnel junction is a breakthrough for further improving the storage density of ferroelectric random access memories. However, the application of these ferroelectric tunnel junctions is limited by high cost of epitaxial perovskite heterostructures, unsatisfactory retention and difficulty of exactly controlling the middle polarization states. In order to overcome the issues, we develop a ferroelectric tunnel junction with smooth ultrathin polycrystalline BiFeO_3_ (BFO) film. Through controlling the polarization state and oxygen vacancy migration using voltage pulses, we demonstrate that voltage-controlled barrier yields a memristive behavior in the device, in which the resistance variations exceed over two orders of magnitude. And we achieve multi logic states written and read easily using voltage pulses in the device. Especially the device is integrated with the silicon technology in modern microelectronics. Our results suggest new opportunity for ferroelectrics as high storage density nonvolatile memories.

## Introduction

As the development of device miniaturization, the demand for non-volatile memory technology has stimulated extensive research. High speed, high storage density and low power consumption have become the new rules to judge a good kind of device^1–3^. Nano ferroelectric devices with superior properties have been emerging as head of next generation nano-electronics, especially the successfully preparation of ultrathin ferroelectric films with good ferroelectricity prompts ferroelectric tunnel junction (FTJ) as new favorite of scientists working on low power consumption devices^4–7^. The newly discussions about ferroelectric domains promote the study and increase competitiveness of ferroelectric devices, basing on controlling the charged domain walls and the ratio of different domains orientations to achieve multilevel data storage^8–11^. The conductance is the functional characteristic of FTJ, while leakage currents are detrimental to the device performance in the ferroelectric capacitors. This property allows using FTJs in non-volatile memory devices that are superior to the existing ferroelectric random access memories^12^. In these FTJs with semiconductor electrodes or two-dimensional material, the barrier height and width can be electrically modulated leading to a greatly enhanced tunneling electroresistance ratio which can be high to 10^9^%^7, 13–15^. All these promote the investigation of multilevel data FTJs (M-FTJs) based on controlling the ratio of different domains orientations, and more than 2 resistance states can be achieved in these M-FTJs. However the M-FTJs with nearly ideal characteristics are mostly demonstrated on epitaxial perovskite heterostructures, which will cause high cost in the preparations^16–21^. Although FTJ with a thin layer of SrTiO_3_ (STO) as an epitaxial template on silicon and an epitaxial film of La_0_._7_Sr_0_._3_MnO_3_ (LSMO) on STO has been reported^7, 19^, it still shows little prospect in the application of M-FTJs. Because the existing M-FTJs with more than 2 resistance states are hard to keep their middle states stable in more than 100 seconds, and hard to exactly control the polarization states, all these limit the application of M-FTJs. The thickness of ultrathin ferroelectric film in FTJ determines that the polarization state is hard to keep stable in a long time. The results of the present investigations suggest that oxygen vacancies are important in the control of ferroelectric barriers, and they are stable in low voltage^22^. Oxygen vacancies in the M-FTJs may promote the application of M-FTJs. In this paper, we create M-FTJs based on oxygen vacancy and polarization controlling barrier height and width. The devices are with polycrystalline ferroelectric films, and the resistance states can be controlled by voltage pulses. Although the ferroelectricity of the polycrystalline ultrathin ferroelectric films is weak, the oxygen vacancies and polarization together exactly promote the formation of middle resistance states. The devices using voltage pulse control are satisfying state reproducibility and integrated with the silicon technology in modern microelectronics. The different resistance states can be distinguished clearly and especially written and read easily. All these will promote the practical application of the multilevel data ferroelectric storage memories.

## Results

**Figure 1:**
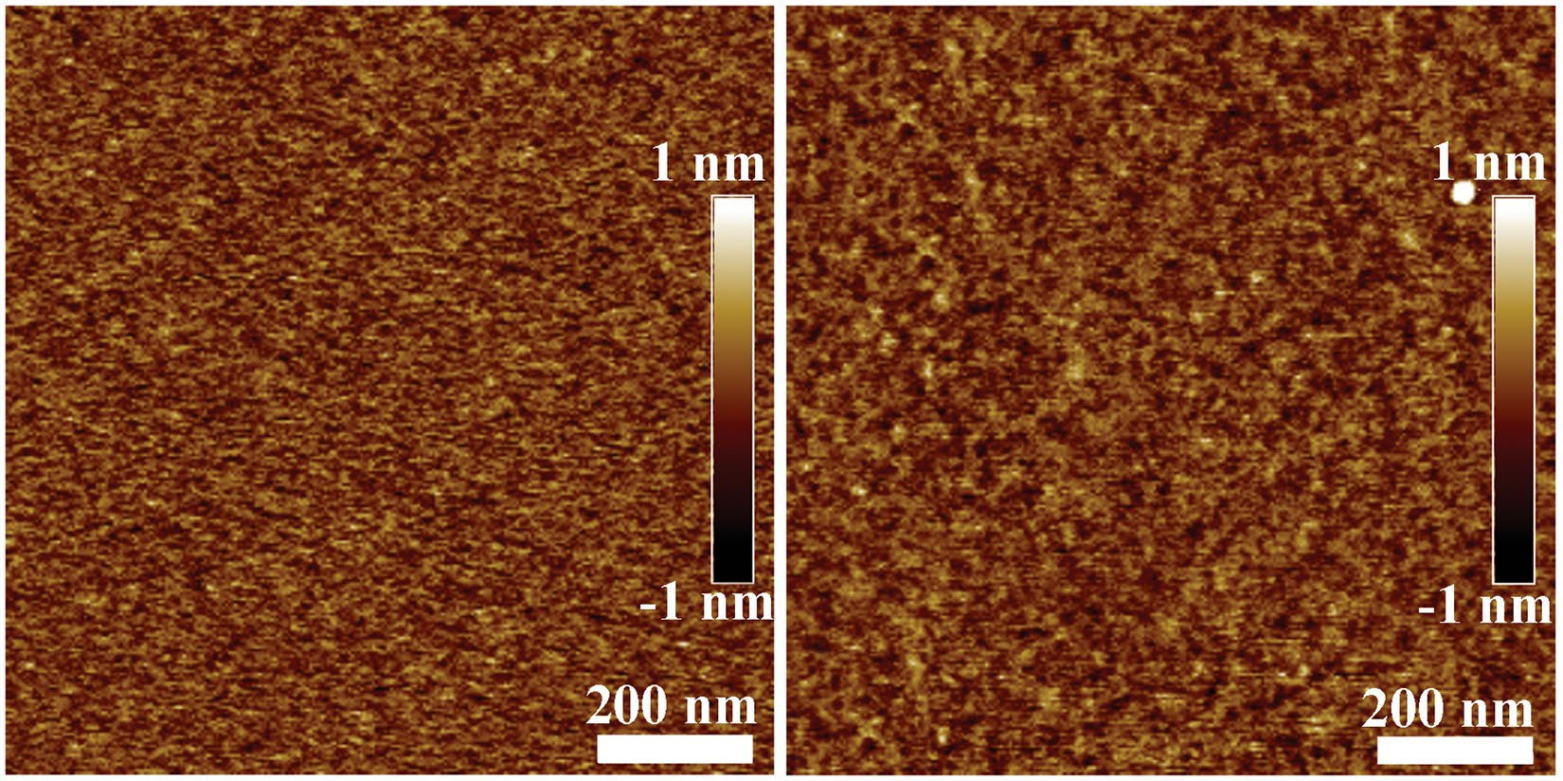
AFM topography of different thick BFO layers on As doped silicon wafer. (**a**) 2 nm; (**b**) 3.5 nm.

**Figure 2:**
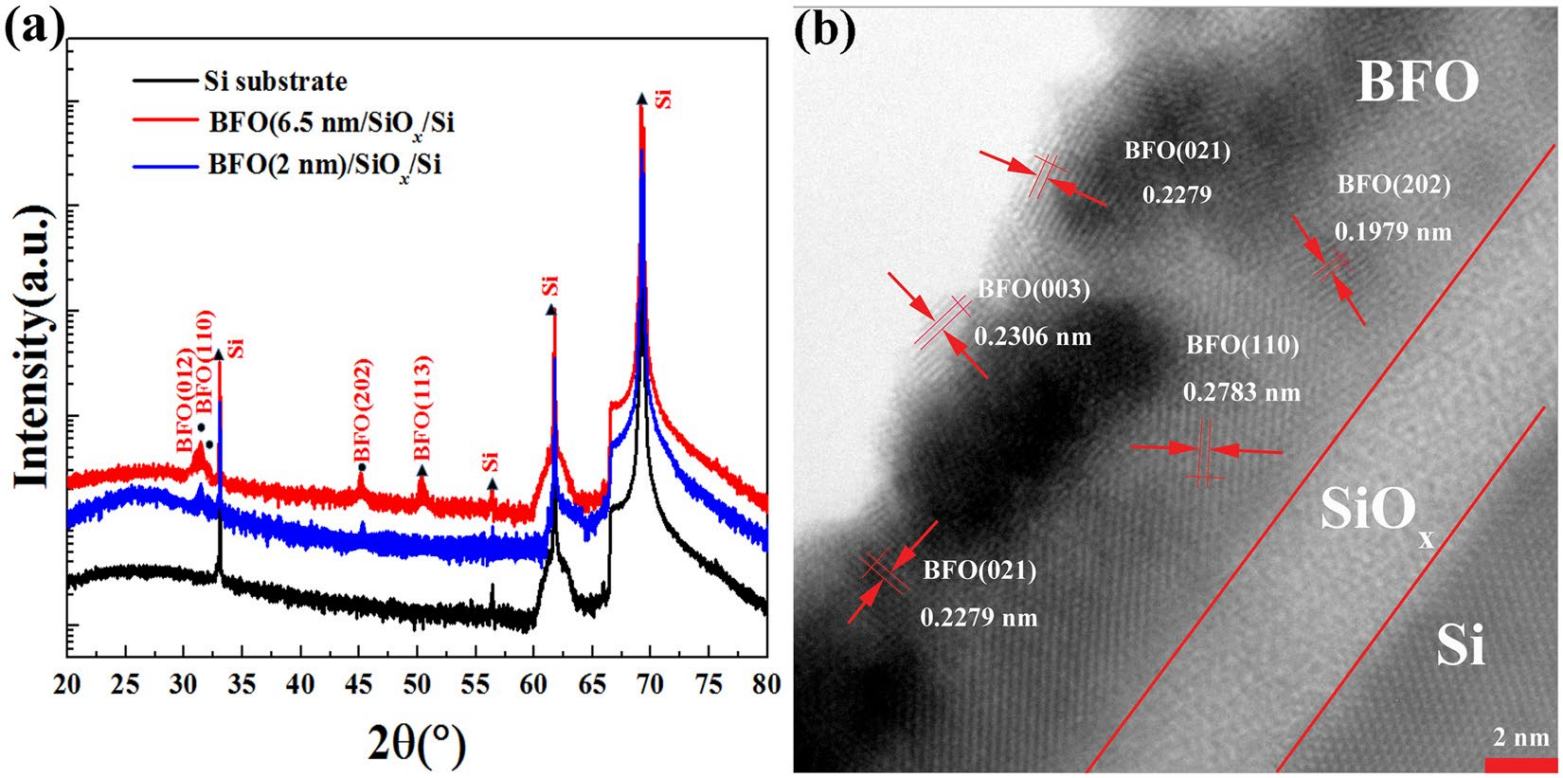
(**a**) XRD patterns of BFO/SiO_*x*_/Si heterostructures and Si substrate at various 2*θ*. (**b**) TEM results of 6.5 nm thick BFO film.

The Atom Force Microscopy (AFM) topography of 2 nm and 3.5 nm thick BFO films is shown in Fig. 1, and the AFM topography of 1 nm, 5 nm and 6.5 nm thick BFO films, and AFM topography of Si wafer are shown in Fig. S1 (in Supplementary Information). The thicknesses of the BFO films are about 1 nm, 2 nm, 3.5 nm, 5 nm and 6.5 nm after deposited 30 s, 60 s, 90 s, 120 s and 150 s using laser molecular beam epitaxy (LMBE). The root-mean-square roughness values of the samples are 0.102 nm, 0.268 nm, 0.152 nm, 0.161 nm, 0.163 nm and 0.172 nm (the deposition time of the samples is 0 s, 30 s, 60 s, 90 s, 120 s and 150 s), and they show that the samples are smooth. Structural characteristics of BFO/SiO_*x*_/Si heterostructures and Si substrate carried out using x-ray diffraction (XRD) in Fig. 2a present peaks at the (012), (110), (202) and (113) planes of BFO films. The Peak Intensity of BFO film is very weak possibly due to very small thickness and the polycrystalline film, and more peaks can be found in the XRD results of 6.5 nm thick BFO film. Between the BFO layer and Si wafer, there is a 3~4 nm thick SiO_*x*_ layer formed in the preparation of the BFO layer because of oxygen diffusion, as shown in Fig. 2b. The (021), (003), (110), (202) planes of BFO film can be found in the TEM results, and the diameters of BFO grains are above 3 nm. The XRD results and the TEM results confirm the presence of polycrystalline BFO films. Pt dot electrodes are fabricated on the BFO films at room temperature as the top electrode, and the substrate is arsenic (As) doped Si wafer which is used as bottom electrode. Obviously, the devices are asymmetry FTJs.

**Figure 3:**
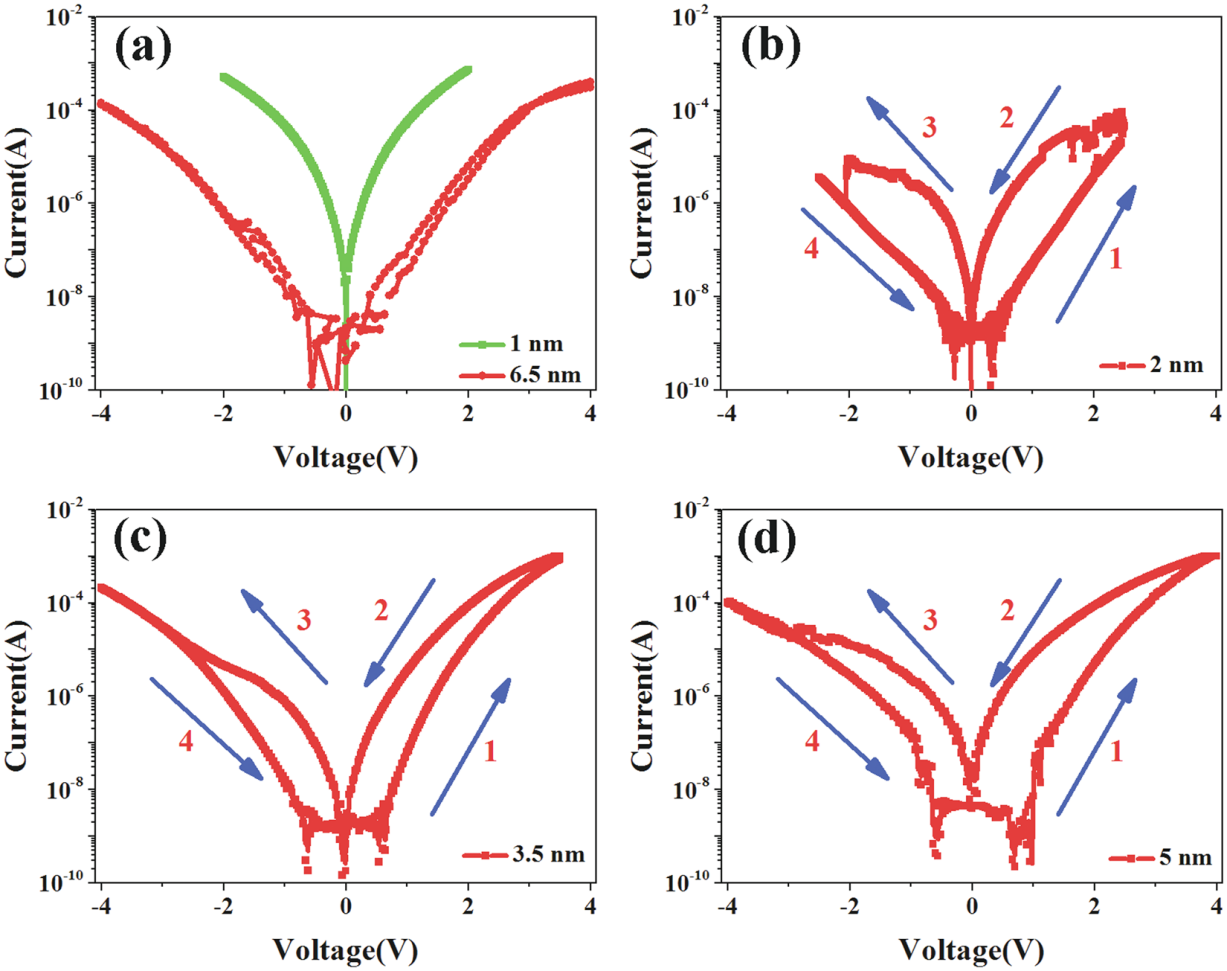
*I*-*V* characteristics of Pt Pt/BFO/SiO_*x*_/Si with different thick BFO films: (**a**) 1 nm and 6.5 nm thick BFO films; (**b**) 2 nm thick BFO film; (**c**) 3.5 nm thick BFO film; (**d**) 5 nm thick BFO film. The blue arrows show the direction of voltage sweeps.

**Figure 4:**
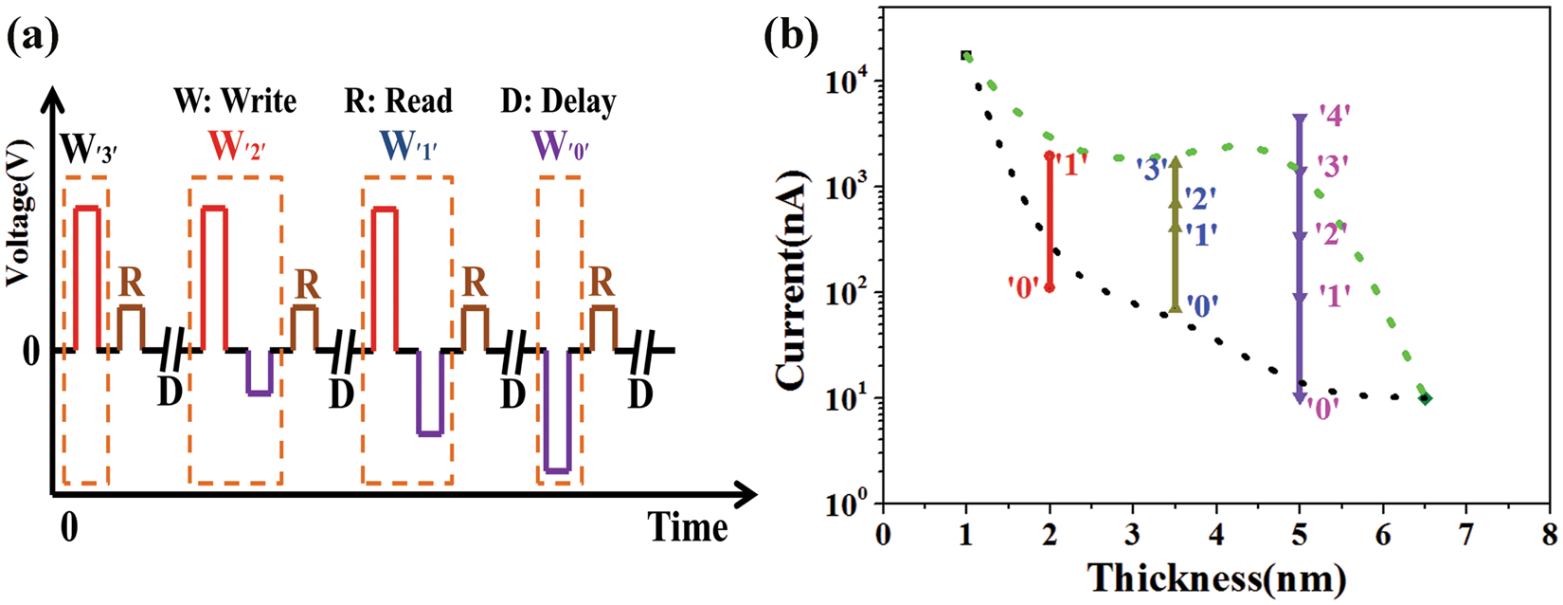
(**a**) The test processes. The voltage pulses applied in the test are about 5 μs. Before writing the middle resistance states (like ‘1’ and ‘2’), a voltage pluse should be applied to set the device at lowest resistantce. (**b**) The current as a function of thickness, all RSs are read at 0.8 V.

As shown in Fig. 3, we swept the voltage in circles (in logs). The sequence of the voltage sweeping is 0 V → positive voltage → 0 V → negative voltage → 0 V. *I-V* curves which are not in logs are shown in Fig. S1 (in Supplementary Information). From the Figs 3 and S2, we can see the switch ratio changes as the BFO thickness at the same read voltage. In order to study the resistance states (RSs) that the devices can achieve at last, we tested the sample as the processes shown in Fig. 4a. It shows that a SET voltage pulse is needed before every write voltage pulse (i.e., before a write voltage pulse of −1 V is applied, a 4 V SET pulse should be applied first), making sure that the initial states are same. SET voltage pulse is applied to get lowest RS of the BFO thin film, negative or positive voltage pulses are applied to get or read different RSs, the results are shown in Fig. 4b, it shows the switch ratio changes with the thickness of BFO film clearly. The current of OFF state (‘0’) decreased as the thickness increasing. The current of ON state decreased at first, and then it increased as the thickness increasing, but it decreased again at last. Finally, we found that the device with 2 nm thick BFO film could achieve 2 RSs, the device with 3.5 nm thick BFO film could achieve 4 RSs, and the device with 5 nm thick BFO film could achieve 5 RSs.

## Discussion

It shows *P-V* loops of the devices with different thick BFO films in Fig. S3 (in Supplementary Information). In the measurement, two tips were connected with Pt electrode and As-doped Si substrate, and the frequency was 1000 Hz. However, these can’t prove the ferroelectricity of BFO films because the tunneling currents are larger than the polarization switching current. So we use PFM to test the ferroelectricity of the BFO films. And according to the PFM test, no local PFM phase hysteresis and amplitude loops can be achieved when the thickness of BFO film is 1 nm or 2 nm. So the BFO film nearly has no ferroelectricity when the BFO film is 1 nm or 2 nm. However, in the test process of 2 nm thick BFO film, the ON state (‘1’) can be achieved by a 2.5 V voltage pulse, and the OFF state (‘0’) can be achieved by a −2.5 V voltage pulse. So the two different RSs of 2 nm thick BFO film are not caused by the polarization switch. Based on the investigations of injection and drift of oxygen vacancies under the application of step voltage in the films, the measured current is identified with the ionic current associated with oxygen vacancies^23, 24^. Considering lots of oxygen vacancies are formed in the SiO_*x*_ layer, they may play important role in the resistance switching. These oxygen vacancies may move into BFO film or out of BFO film in the effect of electric field, it cause the two RSs. When 2.5 V voltage is applied on BFO(2 nm)/SiO_*x*_ films, the electric filed is relatively high. The oxygen vacancy formation energy in SiO_2_ is 0.9 eV and the oxygen vacancy activation energy in the amorphous SiO_*x*_ may be much lower than 0.9 eV^25–27^. Furthermore, the results are consistent with the results of Pt/SrTiO_3_/SiO_*x*_/Si in which two different resistance states can be achieved and the SrTiO_3_ film has no ferroelectricity^28^. These oxygen vacancies which are like fixed positive charges may migrate in the device when large enough external electric field is applied on the device So the two RSs of Pt/BFO(2 nm)/SiO_*x*_/Si are caused by the migration of oxygen vacancies.

**Figure 5:**
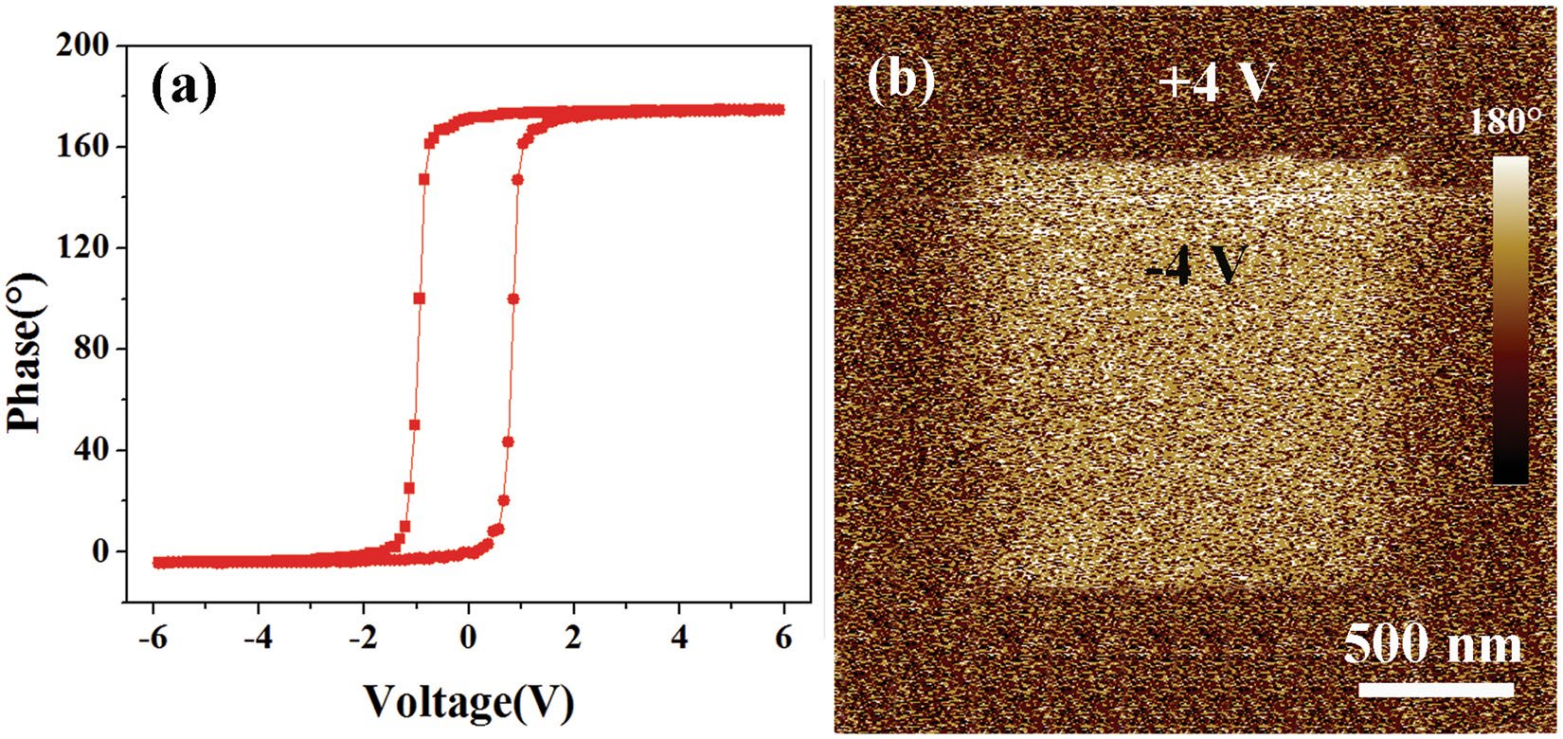
(**a**) Local PFM phase hysteresis loop of 3.5 nm thick BFO thin film. (**b**) Piezoresponse phase image of the 3.5 nm thick BFO film.

**Figure 6:**
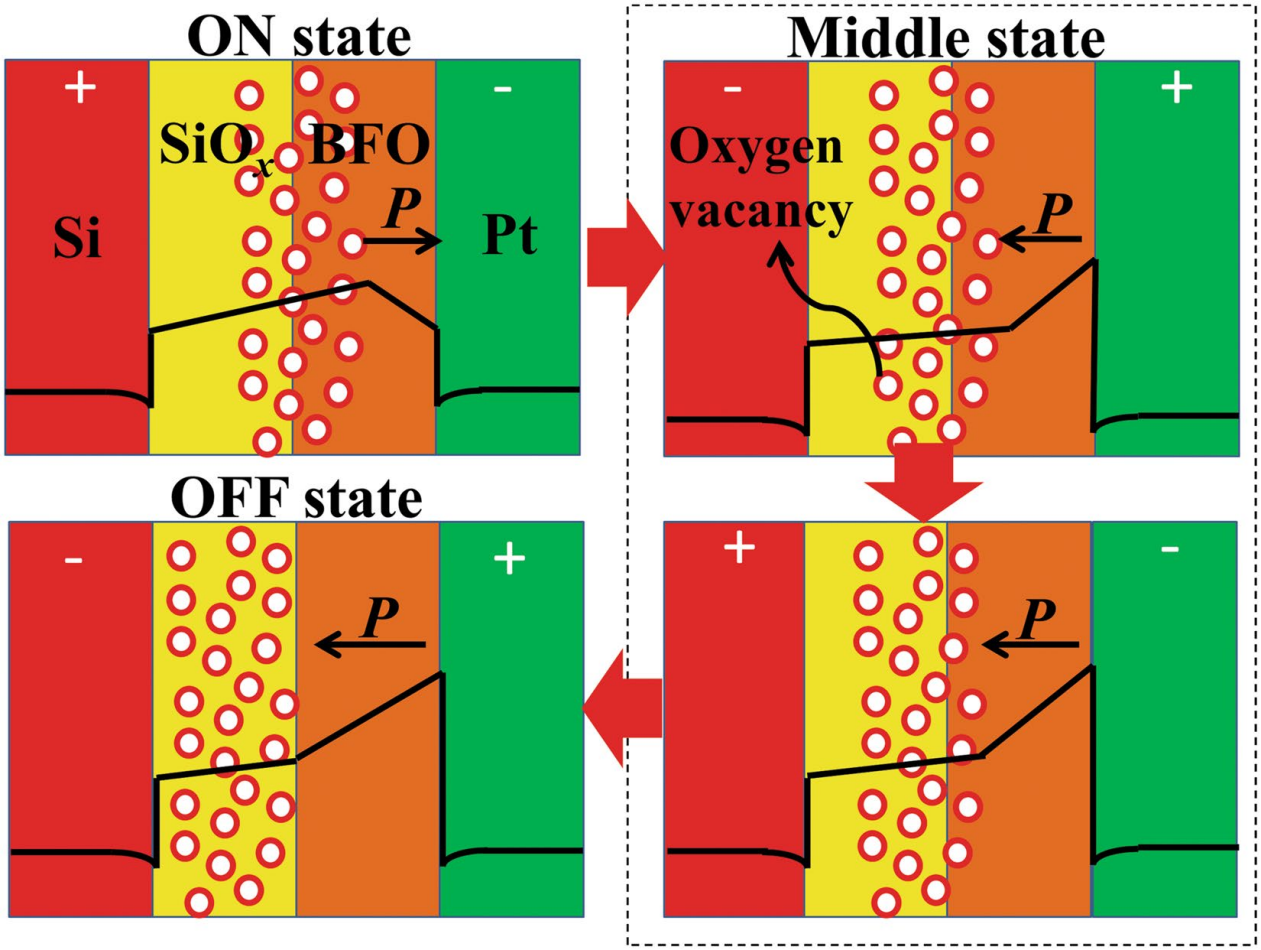
A possible mechanism of ON/OFF state in the device with 3.5 nm thick BFO film.

As the increasing thickness of BFO films, BFO films will have ferroelectricity to tune the RSs with oxygen vacancies together, and the resistance of devices at OFF state is also increasing as the increasing thickness. Figure 5a shows the local PFM phase hysteresis loop of 3.5 nm thick BFO thin film. And a clear ferroelectric response was obtained by using PFM. The electrical poling was performed by scanning at a probe bias of + 4 V over 2.5 μm × 2.5 μm while a reverse voltage of −4 V was applied, leading to the contrast change in the middle of the 1.5 μm × 1.5 μm scanned area, as shown in Fig. 5b. A clear contrast is observed between two distinct regions of opposite polarization. Hence, the clear phase contrast of the 3.5 nm thick BFO film suggests that the film still possesses ferroelectric character. The retention of ferroelectric character measured by PFM is shown in Fig. S1 (in Supplementary Information), the clear contrast between the two distinct regions of opposite polarization can still be observed after 60 h. All these show the ferroelectric character of BFO film has a good retention. In the test process of 3.5 nm thick BFO film, a 4 V voltage pulse was applied on it to get the ‘3’ state, then a −1 V voltage pulse was applied on it to get the ‘2’ state. In order to get the ‘1’ state, a 4 V voltage pulse was applied first, then −2 V voltage pulse was applied. When a −4 V voltage pulse was applied on the film, the ‘0’ state would get at last. Considering the switching between ON state and OFF state, a possible mechanism of ON/OFF states in the device with 3.5 nm thick BFO film is put forward, as shown in Fig. 6. In the effect of positive external electric field, the oxygen vacancies migrate into the ferroelectric film, and the polarization of BFO film which point to Pt decrease a litter, the conductance of BFO film increases. In the effect of negative external electric field, the oxygen vacancies migrate back to the SiO_*x*_ layer, the ferroelectric barrier resumes and the polarization point to SiO_*x*_ layer. Important thing is that the oxygen vacancies at ON state will be stable, because oxygen vacancies need large electric field to migrate at room temperature^27^. So the oxygen vacancies could not migrate in weak external electric field when no illumination is working and the working time of electric field is short. Both the thickness and height of BFO barrier are changing in the device because of the oxygen vacancies migration. In order to prove the mechanism, the current of the device with 3.5 nm thick BFO film has been calculated based on direct tunneling, fowler-nordheim tunneling and thermionic injection in the effect of low electric field. The final current is a combination of the three mechanisms. The calculated results are fit the experimental results, as shown in Fig. S5. (The detailed calculated information is shown in Supplementary Information.) According to the results of calculation, the distance of the oxygen vacancies migration may be very short. The reason may be that the time of voltage pulse applied on the device is very short. Furthermore, the different oxygen vacancies migration distances in the different devices may cause that the current of ON state changed as the thickness increasing, as shown in Fig. 4b.

In the test process of 5 nm thick BFO film, a 4 V voltage pulse was applied on it to get the ‘4’ state, then a −1 V voltage pulse was applied on it to get the ‘3’ state. In order to get the ‘2’ state, a 4 V voltage pulse was applied first, then −2 V voltage pulse was applied. The ‘1’ state would get after the process of a 4 V voltage pulse and a −3 V voltage pulse applied. When a −4 V voltage pulse was applied on the film, the ‘0’ state would get at last. The multilevel date storage mechanism of Pt/BFO(5 nm)/SiO_*x*_/Si device is same to the former mechanism of Pt/BFO(3.5 nm)/SiO_*x*_/Si device. However, when the thickness of BFO layer is too thick, the tunnel current will be hard to read at low read voltage. So the device with 6.5 nm BFO film nearly has no resistance switching phenomenon. In fact, the samples with 3.5 nm and 5 nm BFO films may have more different RSs, but the different RSs caused by different barrier height states should be distinguished clearly and stable in the read process. So we only get the RSs above. In our test, the write processes of the RSs are controlled by voltage pulses, and the time of each voltage pulse applied on the BFO films is constant value about 5 μs. The Pt top electrode used in our work and the write processes using different voltage pulses in our case will not only promote the reliability but also the realization of ultrafast write compared to the conventional multilevel data ferroelectric storage memories written with PFM or CAFM probe sweeping by controlling the write time.

**Figure 7:**
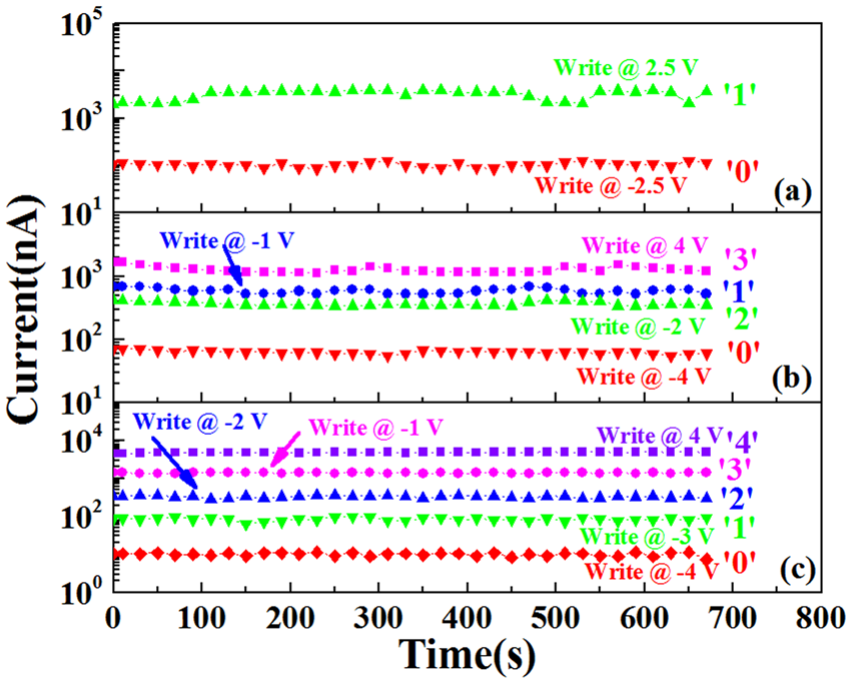
The retention of the Pt/BFO/SiO_*x*_/Si memristors. Current as a function of time. (**a**) The device with 2 nm thick BFO film; (**b**) The device with 3.5 nm thick BFO film; (**c**) The device with 5 nm thick BFO film.

Retention is an important issue for the nonvolatile memory cells. We wrote the 2 states of device with 2 nm thick BFO film, the 4 states of device with 3.5 nm thick BFO film and 5 states of device with 5 nm thick BFO film, and read all the states at 0.8 V to test the retention, on the other hand to test if these states can be distinguished clearly in the read processes. In 700 seconds, the RSs are very stable, as shown in Fig. 7.

In summary, we have fabricated Pt/BFO/SiO_*x*_/Si devices with different thick nonepitaxial BFO ultrathin films, and demonstrated two, four and five states with good retention in them. The write and read processes using voltage pulses are much easier than those in the former reports^7–19^. In especial, the multilevel data ferroelectric storage memory is integrated with silicon technology and cheap for electronic devices, which shows strong promise for future high storage density nonvolatile ferroelectric memory applications.

## Methods

### Device preparation

Different thick polycrystalline BFO films have been grown by laser molecular beam epitaxy (LMBE) on As-doped Si substrates. During deposition of the BFO layer substrate temperature was maintained at 650 °C with chamber oxygen pressure kept at 18 Pa. A SiO_*x*_ layer was formed because of oxygen diffusion during the BFO film deposition. The samples were cooled down to room temperature in oxygen atmosphere at 38 Pa. Pt dot electrodes were fabricated by d.c. sputtering through a shadow mask about 100 μm in diameter at room temperature.

### Electrical characterizations

The film thicknesses have been measured on Filmetrics model F20-UV and F50-UV. Controlled ferroelectric barrier heights have been written using an Agilent B1500A semiconductor device analyzer by applying voltage pulses, and the *I*-*V* measurement characteristic also have been measured on it. The topography of different thick BFO layers on As doped silicon wafers have been performed using an Atom Force Microscopy (AFM). And the piezoresponse AFM images were performed using the AFM with a conductive AFM tip. Cross-sectional TEM image of BFO/SiO_*x*_/Si heterostructure has been performed using Transmission Electron Microscope (TEM).

## Electronic supplementary material

Supplementary Information
