# Supplementary material for: Investigation of multilevel data storage in silicon-based polycrystalline ferroelectric tunnel junction
Source: Sci Rep. 2017 Jul 3;7:4525. doi: 10.1038/s41598-017-04825-z (PMC5495759; doi:10.1038/s41598-017-04825-z)
Supplement: Supplementary file 1 — Supplementary Information [file 41598_2017_4825_MOESM1_ESM.doc]

**Investigation of multilevel data storage** **in silicon-based polycrystalline ferroelectric tunnel junction**

Pengfei Hou,^1,2,3^ Jinbin Wang^1,2,3^ and Xiangli Zhong^1,2,3^

^1^ *School of Materials Science and Engineering, Xiangtan University, Hunan Xiangtan 411105, China*

^2^*Hunan Provincial National Defense Key Laboratory of Key Film Materials & Application for Equipment, Xiangtan University, Hunan Xiangtan 411105, China*

*^3^ Key Laboratory of Low-dimensional Materials and Application Technology, Xiangtan University, Hunan Xiangtan 411105, China*

**Corresponding Author**

Author to whom correspondence should be addressed. Tel: 86-731-58293030; Fax: 86-731-58298119; Electronic mail: [*jbwang@xtu.edu.cn*](mailto:jbwang@xtu.edu.cn)*.*

**Equal Contribution**

These authors contributed equally to this work.

**Supplementary Note 1**


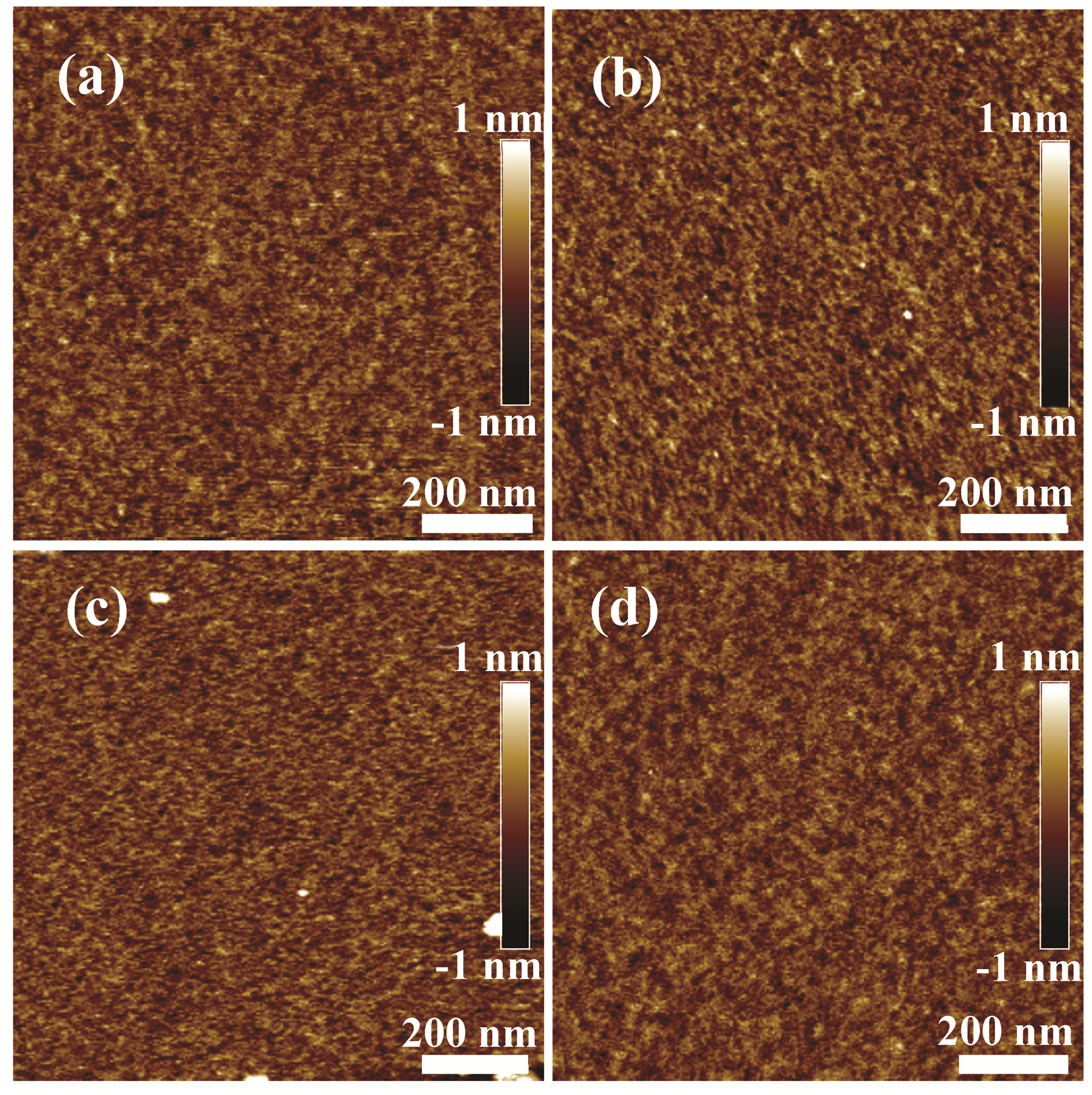


**Figure S1.** The AFM topography of 1 nm (a), 5 nm (b) and 6.5 nm (c) thick BFO films, and Si wafer (d).


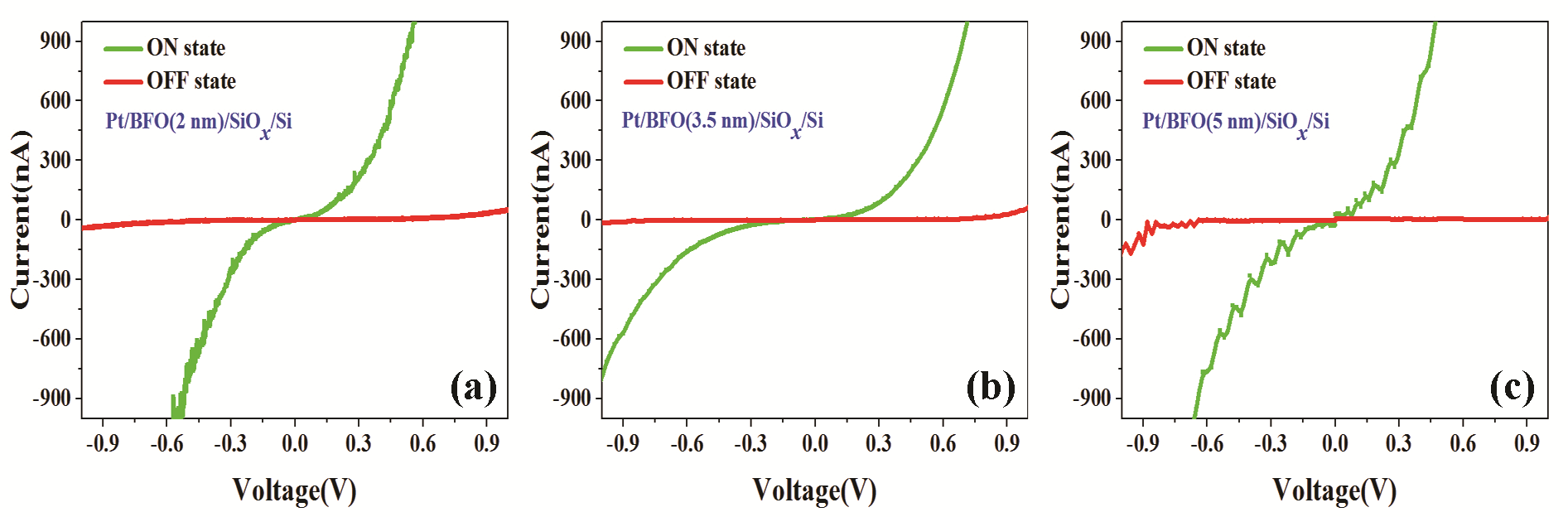


**Figure S2**. *I-V* curves of Pt/BFO/SiO*_x_*/Si device: (a) 2 nm thick BFO film; (b) 3.5 nm thick BFO film; (c) 5 nm thickness BFO film.


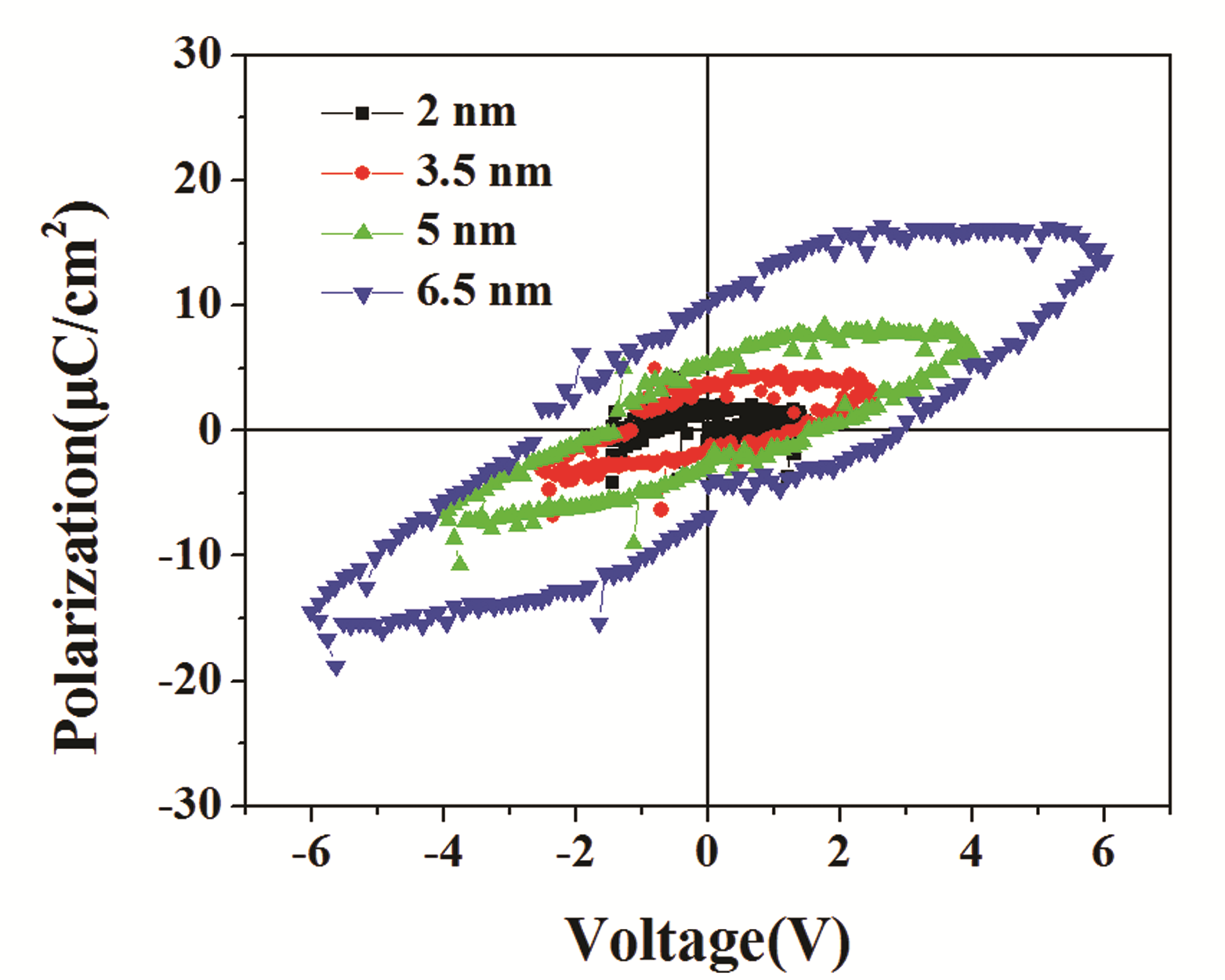


**Figure S3**. *P-V* loops of the devices with different thick BFO films.

The ferroelectric character retention of 3.5 nm thick BFO film by PFM has been provided. As shown in Figure S4, the clear contrast between the two distinct regions of opposite polarization can still be observed after 60 h.


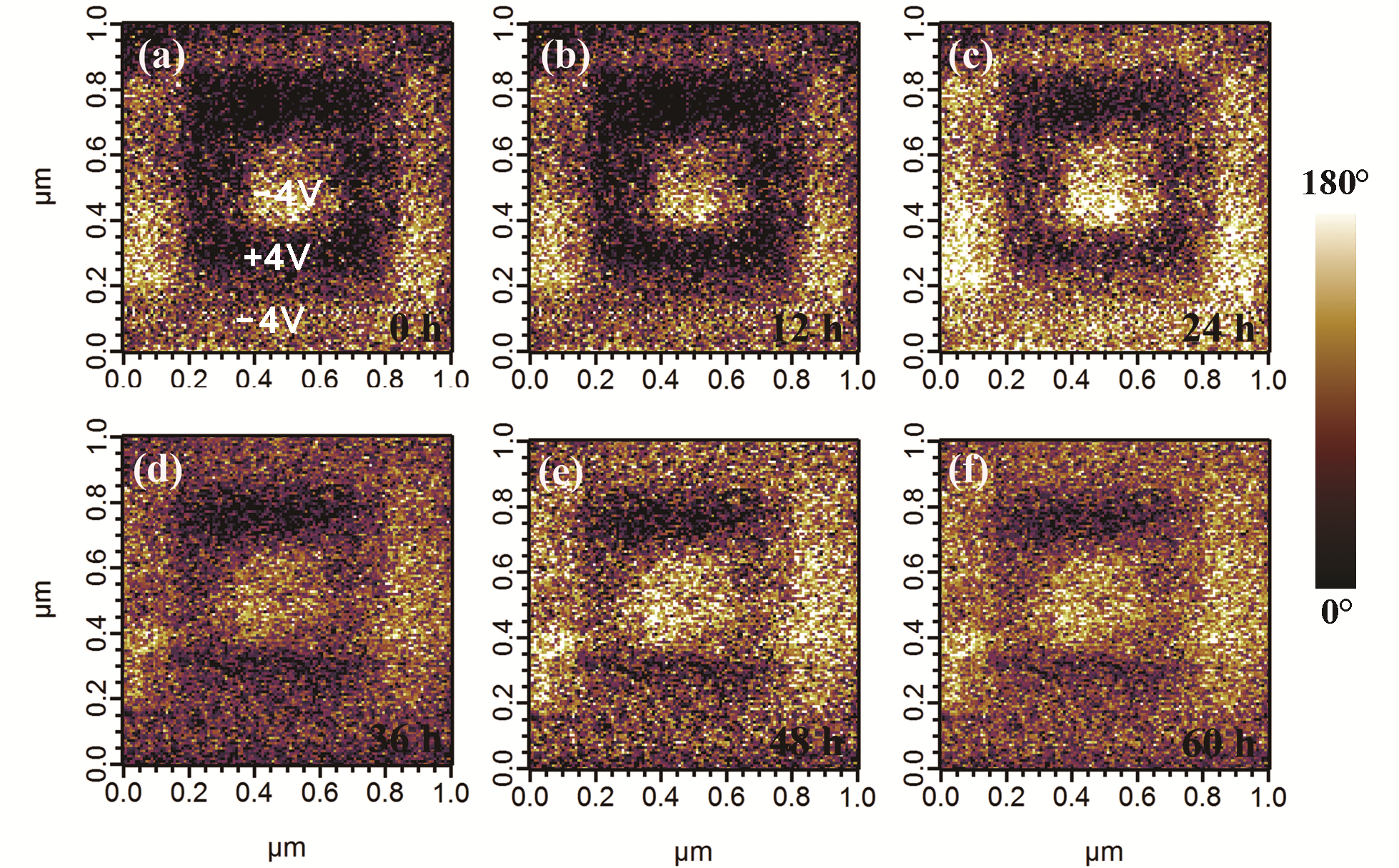


**Figure S4**. Phase-switching images of BFO film. (a) At first. (b)After 12 h. (c) After 24 h. (d)

After 36 h. (e) After 48 h. (f) After 60h.

**Supplementary Note 2**

**Detailed calculated information**

The current of the device with 3.5 nm thick BFO film based on direct tunneling, Fowler-Nordheim tunneling and thermionic injection has been calculated as followings.

The BFO with a polarization *P* and a static permittivity *ε*_stat_ is sandwiched between SiO*_x_* and Pt with different Thomas-Fermi screening lengths *l*_1_ and *l*_2_. A voltage *V* is applied on the device and the resulting current *I* through the SiO*_x_*–BFO-Pt heterostructure is calculated in dependence on the ferroelectric polarization direction. The polarization *P* is perpendicular to the film surface, either pointing toward the contact where the voltage is applied (*P* > 0) after switching with a negative voltage or away from it (*P* < 0) after switching with a positive voltage.

The screening charge density *Q*_s_, given by

$Q_{s}=\frac{Pd}{\varepsilon_{stat}(\frac{l_{1}}{\varepsilon_{M,1}}+\frac{l_{2}}{\varepsilon_{M,2}})+d}$ . (1)

The depolarization field *E*_depol_ and the change in the potential barriers 2∆Φ_i_ on polarization reversal inside the ferroelectric can be calculated from Thomas-Fermi screening.

$E_{depol}=-\frac{(P-Q_{s})}{\varepsilon_{0}\varepsilon_{stat}},$ (2)

$\Delta\Phi_{i}=\frac{l_{i}Q_{s}}{\varepsilon_{0}\varepsilon_{m,i}}e,i=1,2,$ (3)

The potential barrier without image force lowering is then given by

$\Phi_{B,i}=\Phi_{i}\pm\Delta\Phi_{i}$ (4)

where Φ_1,2_ is the barrier without polarization which might be different for different electrode. The upper sign (+) applies for Φ_1_ (the barrier at the interfaces between metal SiO*_x_* and the ferroelectric) and the lower sign (-) for Φ_2_ (the barrier at the interfaces between metal Pt and the ferroelectric). The potential barrier Φ_B_ is the energy barrier which the electrons must overcome during transport across the SiO*_x_*-BFO-Pt heterostructure, i.e., Φ_B,1_ for *V* > 0 and Φ_B,2_ for *V* < 0.

Applied field *E_ap_* = -*V*/*d*, the field due to band alignment *E*_band_, $E_{band}=\frac{\Phi_{2}-\Phi_{1}}{ed}$.

However, here, we focus on the polarization dependence and introduce it by employing a model proposed by Zhuravlev et al.^2^ We used the direct tunnel current density *j_DT_* given by Gruverman et al.^3^

(5)

Where , , *m*_e,ox_ being the effective tunneling electron mass.

Thermionic injection describes the current which is due to charge carriers which overcome the potential barrier by thermal energy. The barrier height is lowered by image force lowering, called the Schottky effect.^4^ The thermionic injection current density *j*_Schottky_ can be described for sufficiently high voltages.

$j_{Schottky}=A^{**}T^{2}\exp\left[-\frac{1}{k_{B}T}\left(\Phi_{B}-\sqrt{\frac{e^{3}E}{4\pi\varepsilon_{0}\varepsilon_{ifl}}}\right)\right]$ (6)

Where *A^**^*the effective Richardson’s constant, and *ε*_ifl_ the permittivity of the ferroelectric responsible for image force lowering.

Fowler-Nordheim tunneling (FNT) is tunneling across a triangular-shaped potential barrier, which is formed by applying an electrical field *E* to a rectangular or trapezoidal barrier.^5^ FNT is basically the same physical phenomena as direct tunneling, but in a different voltage regime.

$j_{FN}=\frac{e^{3}m_{e}}{8\pi hm_{e,ox}\Phi_{B}}E^{2}\exp\left[-\frac{8\pi\sqrt{2m_{e,ox}}}{3he}\frac{\Phi_{B}^{\frac{3}{2}}}{E}\right]$ (7)

In both the latter mechanisms the electric field *E* is the field responsible for band tilting. The parameters corresponding to BFO (*d* = 3.5 nm, *P* = 3 μC/cm^2^, *ε*_stat_ = 200, *ε*_ifl_ = 10, *m_e_,*_ox_ = m_e_, and *A^**^*= 10^6^ A m^-2^ K^-2^) and its interface with SiO*_x_* (*l_1_* = 0.8Å, Φ_1_ = 0.915 V, *ε*_M,1_ = 8) and Pt [*l_2_* = 0.55 Å, Φ_2_ = 0.9 V, and *ε*_M,2_ = 2] were used. When the device is at ON state, the oxygen vacancies will migrate to the interfaces of SiO*_x_* and BFO layers, so the d will be smaller, and we assumed that the distance of oxygen vacancy migration is about 0.7 nm. The final current is a combination of the three mechanisms. However, direct tunneling current is prominent at low voltage and Fowler–Nordheim tunneling dominates at large voltage. As the barrier thickness increases, the direct tunneling current decreases exponentially and the current due to thermionic emission dominates. The calculated result is shown in figure S5, in which the current density has been changed to current and the diameter of the electrode is about 100 μm. The calculated results are fit the experimental result. So the current measured is a tunnel current.

**
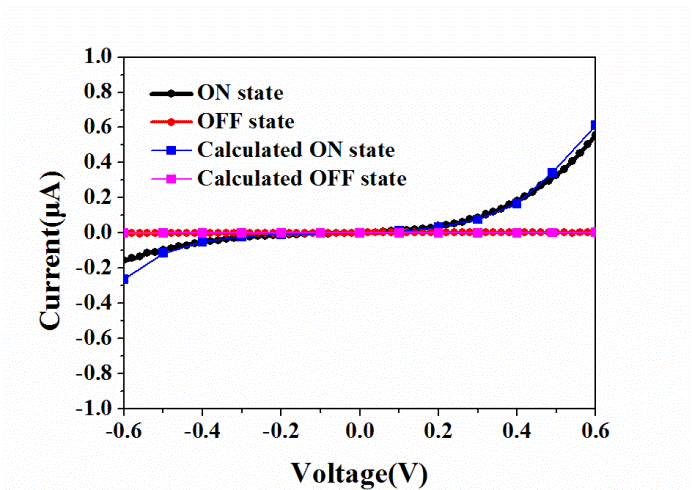
**

**Figure S5**. Experimental and calculated *I-V* curves of the device with 3.5 nm thick BFO film.

**Supplementary Note 3**

When a slight SrRuO_3_ (SRO) is between the SiO*_x_* and BFO film (5 nm), the *P-V* loop may be much beautiful, but the *P*r value is still smaller than the *P*r value of BFO epitaxial film, as shown in the figure S6. So the curve shape may be affected by the n-Si electrode,^1^ and the *P*r value may be affected by the n-Si electrode, polycrystalline film and thickness of BFO film.


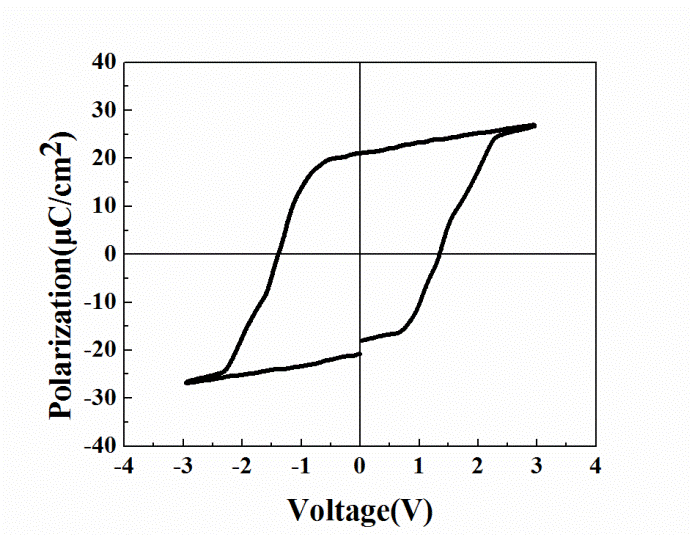


**Figure S6**. *P-V* curve of Pt/BFO/SrRuO_3_(SRO)/SiO*_x_*/Si device. The polycrystalline BFO film is about 5 nm, and the polycrystalline SRO film is about 7 nm.

**References**

^1^ Yuan, S., Wang, J., Zhong, X., Huang, J. and Zhou, Y. Size effect on the ultrathin ferroelectric film directly grown on silicon for electronic devices. *RSC Adv.* 3, 24362(2013).

^2^ Zhuravlev, M. Y., Sabirianov, R. F., Jaswal, S. S., and Tsymbal, E. Y. Giant electroresistance in ferroelectric tunnel junctions. *Phys. Rev. Lett.* 94, 246802 (2005).

^3^ Gruverman, A., et al. Tunneling electroresistance effect in ferroelectric tunnel junctions at the nanoscale. Tsymbal, *Nano Lett.* 9, 3539 (2009).

^4^ Sze, S. M., Ng, K. K. Physics of semiconductor devices. *John wiley & sons* (2006).

^5^ Fowler, R. H. and Nordheim, L. Electron emission in intense electric fields. *Proc. R. Soc. London, Ser. A* 119, 173 (1928).
